# Europium Silicide – a Prospective Material for Contacts with Silicon

**DOI:** 10.1038/srep25980

**Published:** 2016-05-23

**Authors:** Dmitry V. Averyanov, Andrey M. Tokmachev, Christina G. Karateeva, Igor A. Karateev, Eduard F. Lobanovich, Grigory V. Prutskov, Oleg E. Parfenov, Alexander N. Taldenkov, Alexander L. Vasiliev, Vyacheslav G. Storchak

**Affiliations:** 1 National Research Center “Kurchatov Institute”, Kurchatov Square 1, Moscow 123182, Russia

## Abstract

Metal-silicon junctions are crucial to the operation of semiconductor devices: aggressive scaling demands low-resistive metallic terminals to replace high-doped silicon in transistors. It suggests an efficient charge injection through a low Schottky barrier between a metal and Si. Tremendous efforts invested into engineering metal-silicon junctions reveal the major role of chemical bonding at the interface: premier contacts entail epitaxial integration of metal silicides with Si. Here we present epitaxially grown EuSi_2_/Si junction characterized by RHEED, XRD, transmission electron microscopy, magnetization and transport measurements. Structural perfection leads to superb conductivity and a record-low Schottky barrier with *n*-Si while an antiferromagnetic phase invites spin-related applications. This development opens brand-new opportunities in electronics.

Integrated-circuit scaling faces fundamental restrictions in the areas of design, manufacturing, energy and physical space^1^. Information technologies have entered the era of material limited device scaling^2^: basic materials of electronics have been extended to their performance limits. Likewise, emerging computing technologies pose further challenges to materials research. In particular, front end processes shift focus onto contacts in nanoscale devices^2^, thus emphasizing the importance of precise control over the structure and composition of the metal-semiconductor (MS) interface^3^.

Both reduced dimensionality and device scaling lead to soaring current densities raising problems of heat dissipation and electromigration. Interdiffusion and uncontrolled chemical reactions affect the properties of materials and interfaces, thus destroying device functionality. The Schottky barrier height (SBH), the most important characteristic of an MS interface, exhibits sharp dependence on the interfacial chemistry^4^. In particular, Si – a workhorse of modern electronics – reacts with most metals to form silicides^5^.

Nowadays, metal silicides are an integral part of microelectronics being used as ohmic and Schottky barrier (SB) contacts, interconnects, gate electrodes or diffusion barriers^6^. Compatibility with Si technology, low resistance, suppression of electromigration, good contacts to other materials ensure numerous applications of silicides. In particular, the self-aligned silicide (SALICIDE) technology free of lithographic patterning processes is commonly employed for manufacturing ultra-large-scale integration devices^7^. Mid-gap silicides TiSi_2_, CoSi_2_ and NiSi are most popular materials due to low resistivity but scaling to ultra-small gate lengths and junction depths is challenging: issues like phase purity and Si consumption become increasingly important^5^,^6^,^8^. When reduced to nanoscale, silicides make a frontier research subject: they form nanodots and nanowires with appealing properties^5^,^6^,^9^,^10^, are employed as contacts to Si nanowires^11^,^12^,^13^ and integrate Si technology with prospective materials like graphene^14^.

Rare-earth (RE) silicides make a special class of materials with a low SBH to *n*-Si^15^. This property ensures their use as source/drain terminals in the SB metal-oxide-semiconductor field-effect transistors (MOSFET) technology^16^ combining advantages of low parasitic resistance, small junction depth, high channel mobility, low temperature processing. The device scaling to sub-10 nm gate lengths requires atomically abrupt junctions as well as the epitaxial quality of silicide layers alleviating the problem of grain boundaries, improving thermal stability and uniformity^5^. Among RE silicides, crystalline YbSi_2−x_ and ErSi_2−x_ are leading candidates for SB-MOSFET due to their lowest values of SBH (0.27–0.28 eV, to compare with ~0.6 eV for widely used transition metal mid-gap silicides) after thermal annealing^17^,^18^,^19^ which can be further reduced by segregation of impurities at the silicide/Si interface^20^. Nevertheless, the technology demands contact materials free of manufacturing problems such as non-stoichiometry and with yet lower SBH.

Additional requirements are imposed on the contact material if it is designed to be compatible with spintronic applications. Silicon spintronics is an emerging set of energy-efficient information technologies implementing spin functionality in Si^21^,^22^. Injection of spin-polarized carriers into a semiconductor is demonstrated from magnetic semiconductors^23^, half-metals^24^, and metals through insulating tunnel barriers^25^,^26^,^27^,^28^,^29^ or Schottky-tunnel-barrier contacts^30^. Ohmic contacts are not functional without spin pumping^31^. Thus, SBH is a crucial parameter for metal/semiconductor spin injection^32^,^33^. Although transient femtosecond spin current can be induced in a non-magnetic material^34^, spin injection requires magnetic contacts. In general, any magnetic order suppresses spin scattering. Ferromagnetic silicides MnSi^35^ and Fe_3_Si^36^ are effective spin injectors. On the other hand, antiferromagnetic (AFM) contacts are also functional in spintronics applications as they support spin currents and better suited for using as spin detectors than ferromagnets^37^. AFM buffer layers may enhance spin transfer efficiency from a ferromagnet^38^.

Here, we propose stoichiometric europium silicide EuSi_2_ as a new multifunctional material for contacts with Si in nanoelectronics. We demonstrate that epitaxial EuSi_2_/Si junction is easy-to-manufacture and free of alien phases. The quality of the contact is confirmed by a number of techniques. In particular, transmission electron microscopy reveals the atomically sharp EuSi_2_/Si interface. The SBH of the EuSi_2_/*n*-Si junction (0.21 eV) is determined to be the lowest among all silicides suggesting its use in the SB-MOSFET technology. The AFM phase of EuSi_2_ invites its applications in spintronics.

## Results and Discussion

Europium (II) compounds are famous due to a wide range of electrical, magnetic and optical properties but the Eu-Si system and, in particular, EuSi_2_ are far from being well-studied. EuSi_2_ crystallizes in the tetragonal α-ThSi_2_ structure type (*I41/amd* space group) with lattice constants 4.304, 4.304 and 13.65 Å^39^. Europium is highly reactive and its reaction with Si does not require high temperature: EuSi_2_ appears as a common side product of EuO growth on Si surfaces^40^,^41^. At the same time attempts to grow epitaxial films of EuSi_2_ have been unsuccessful: resulting in nanoislands and/or polycrystalline films^42^. Studies of the early stage of the silicide formation on Si (111) reveal that Si is the dominant diffusion species^43^ which is common for disilicides^6^. The outcome of the reaction is probably governed by the Si diffusion rate which strongly depends on the substrate temperature. Therefore, in our studies of EuSi_2_ growth on Si (001) substrate we pay special attention to fine tuning of temperature and Eu flux.

**Figure 1:**
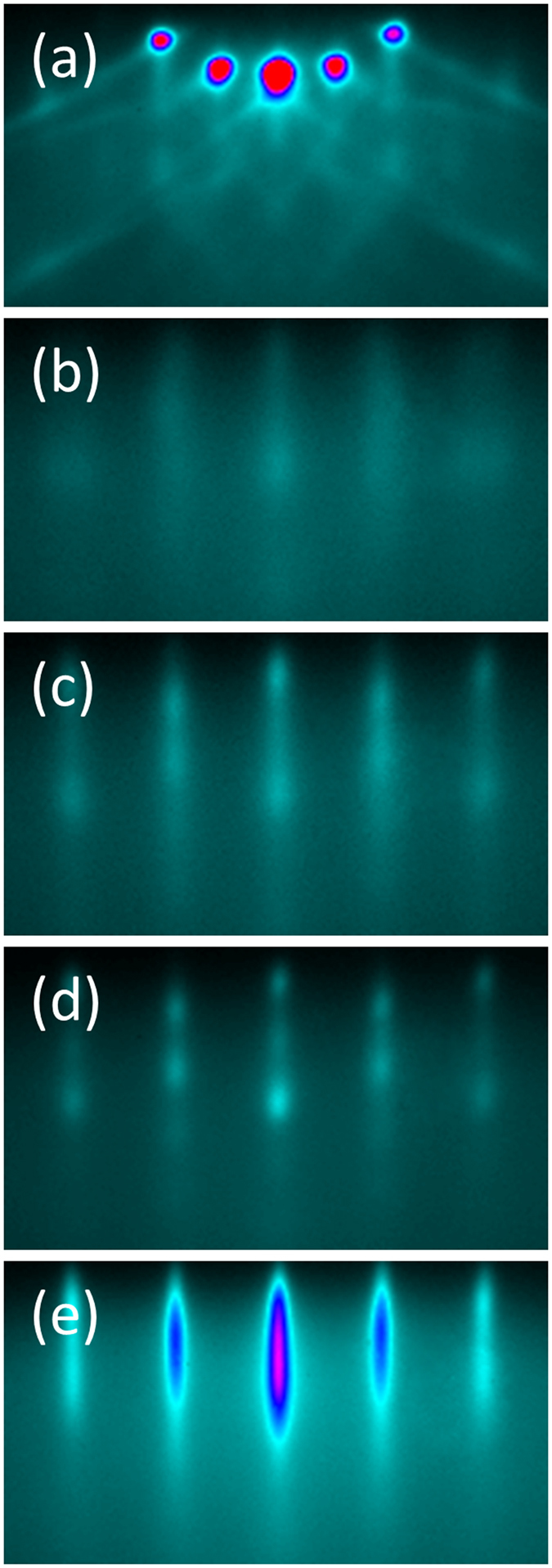
RHEED images along the [110] azimuth of silicon substrate (azimuth [100] for the grown EuSi_2_ film): (**a**) Initial clean Si surface with reconstruction 2 × 1 + 1 × 2. (**b**) About 30 Å of EuSi_2_ grown on Si at 400 °C. (**c**) About 50 Å of EuSi_2_ grown on Si at 400 °C. (**d**) After the growth at 400 °C, about 530 Å of EuSi_2_ on Si. (**e**) After high-temperature growth at 560 °C, about 560 Å of EuSi_2_ on Si.

To control the state of the surface during the growth we routinely employ reflection high-energy electron diffraction (RHEED) images along the [110] azimuth of the Si substrate. The surface of the substrate is prepared by heating up to 950 °C (according to pyrometer readings) to remove the natural surface SiO_2_ layer. The resulting surface exhibits 2 × 1 + 1 × 2 diffraction pattern (Fig. 1a). Then, the substrate kept at a temperature of 400 °C is exposed to a constant Eu pressure of 1.5·10^−8^ Torr coming from a Knudsen cell kept at 470 °C. It corresponds to adsorption-controlled growth (also known as MBE distillation) with an average rate 3 Å/min. It implies that the Eu flux can be varied widely without much effect on the growth outcome as long as the growth regime remains the same. RHEED images reveal a sequence of growth stages. It is common for RE silicides to self-organize and form a number of high aspect ratio nanowires when sub-monolayer amounts of metal are deposited on the surface. Also, a number of surface phases are known for RE metals on Si, including those for Eu^40^,^44^. Three of them are observed at the beginning of the growth: successive surface superstructures 1 × 2 + 2 × 1, 1 × 5 + 5 × 1 and 1 × 3 + 3 × 1 are followed by formation of wide stripes on the RHEED image (Fig. 1b). Next, the stripes become thinner indicating improvement of the crystalline quality; the set of reflections gradually transforms to one that can be attributed to a single crystalline EuSi_2_ layer (Fig. 1c). Pronounced intensity modulation along the stripes at the later stages of the growth (Fig. 1d) is a fingerprint of surface roughening. However, addition of another short high-temperature (560 °C) growth stage results in smoother surface (Fig. 1e). Films with thickness up to 560 Å have been manufactured. The lateral lattice parameter determined from the distance between streaks in the RHEED pattern is equal to 4.34 ± 0.05 Å. A capping layer of SiO_x_ with a thickness of 200 Å is deposited on top of EuSi_2_ to ensure its protection from the air.

Notice that our procedure is not very different from that employed in ref. 42 but the remarkable change of the outcome (single crystal instead of polycrystalline film) originates from a meticulous optimization of the growth conditions. The stability of the EuSi_2_ structure probably plays a great role in the easy formation of the epitaxial silicide film: similar reaction of Sr with Si substrate results in polycrystalline SrSi_2_ despite Sr being an equally active metal and very similar ionic radii of Eu(II) and Sr. Since the synthesis requires relatively mild conditions a reduced thermal budget is expected for its technological implementation. Although non-stoichiometry is often observed in rare-earth disilicides the problem is significant for hexagonal and orthorhombic structures while the tetragonal phase (like EuSi_2_) is characterized by a composition close to stoichiometric. As for our particular system EuSi_2_/Si(001), a synchrotron radiation study of EuSi2 nanoislands and polycrystalline films^45^ shows that the product of reaction between Eu and the Si(001) substrate has the EuSi_2_ stoichiometry.

**Figure 2:**
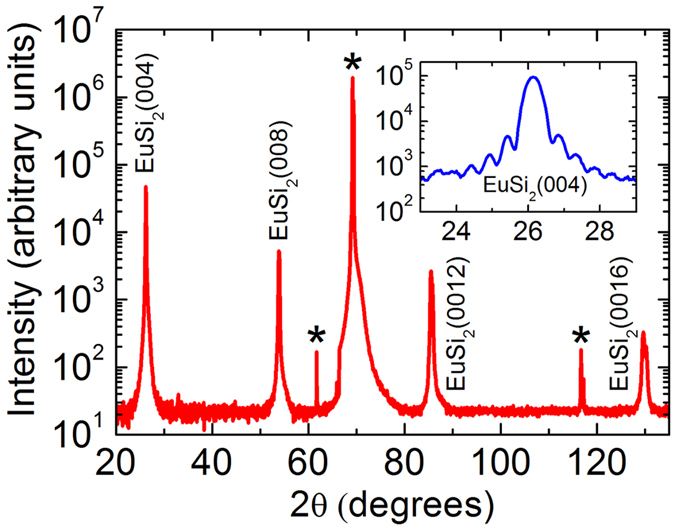
θ–2θ X-ray diffraction scan of the EuSi_2_/Si junction (56 nm of the silicide). The spectrum reveals allowed peaks of EuSi_2_, namely (004), (008), (0012) and (0016). Stars (*) denote peaks from the Si substrate. No extrinsic peaks are detected. Inset: thickness fringes around EuSi_2_ (004) reflection for a 20 nm film.

The epitaxial quality of the film is confirmed by X-ray diffraction (XRD) studies: Fig. 2 shows a typical θ–2θ XRD scan displaying peaks (004), (008), (0012) and (0016) from EuSi_2_ as well as peaks from the substrate. No other phases are detected. All EuSi_2_ peaks correspond to the same orientation with the *c*-axis orthogonal to the surface. EuSi_2_ crystallites with such orientation are also observed in XRD spectra of polycrystalline EuSi_2_^42^ however accompanied by a number of other orientations. A lattice parameter of 13.633 ± 0.006 Å in the direction orthogonal to the EuSi_2_/Si interface is determined from the location of reflections in the θ–2θ scan.

Thickness fringes are observed for the EuSi_2_ (004) reflection (see inset in Fig. 2). This characteristic feature of x-ray diffraction is a result of the wave interference due to reflections at the interfaces, both top and bottom. Taking into account the value of the x-ray wave length (1.5418 Å), the observation of the thickness fringes is a fingerprint of sharp interfaces; otherwise the reflected waves cannot maintain the coherence and thickness fringes would not show up.

**Figure 3:**
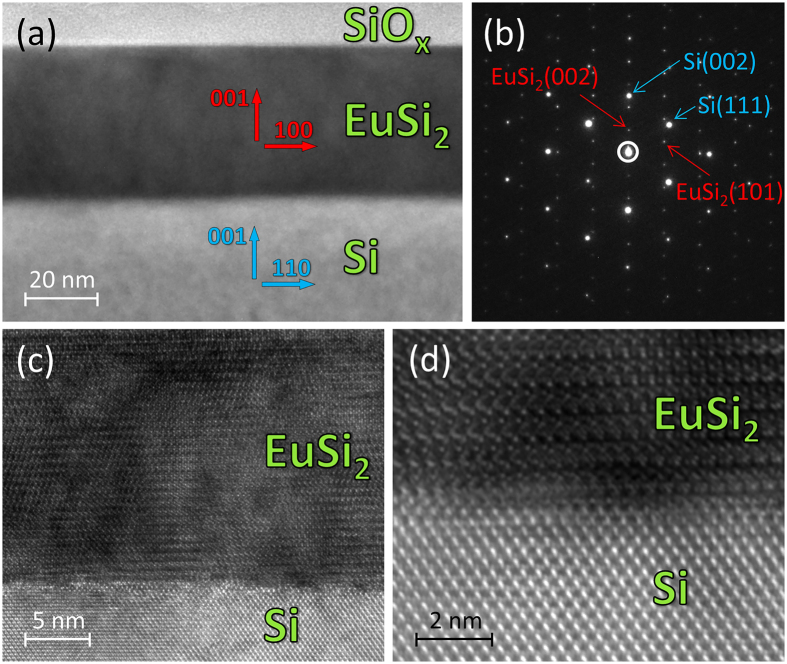
Microscopic structure of the EuSi_2_/Si junction. (**a**) Low-magnification cross-sectional bright-field TEM image of the 56 nm EuSi_2_ film on Si protected by SiO_x_ viewed along the [110] zone axis of the Si substrate and showing the absence of side products. (**b**) Selected area electron diffraction pattern of EuSi_2_ superimposed with that of Si revealing their relative orientation. (**c**) Medium-magnification cross-sectional bright-field TEM image of the EuSi_2_/Si interface showing out-of-phase boundaries in the film. (**d**) High-resolution cross-sectional bright-field TEM image demonstrating atomic structure of the EuSi_2_/Si interface.

The carrier injection efficiency of the structure depends on the properties of the EuSi_2_/Si interface rather than on the overall quality of the film. Thus, a study of the films with transmission electron microscopy (TEM) techniques becomes indispensable. A bright field TEM image of the EuSi_2_ film on Si (001) is shown in Fig. 3a. Even at low magnification the interface looks sharp and smooth, without any unevenness like precipitates with facets parallel to (111) Si planes. Our experiments result in a very smooth top surface of the film in strong contrast to polycrystalline EuSi_2_ films of previous attempts^42^. The selected area (electron) diffraction pattern (SADP), shown in Fig. 3b, certifies that EuSi_2_ adopts a tetragonal crystal lattice with lattice parameters *a* = 4.3 Å and *c* = 13.6 Å, quite close to those known in the literature^39^ and those determined in our RHEED and XRD studies. The orientation relationships derived from the SADP are:


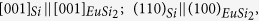


corroborating the RHEED and XRD data. It means that the lattice mismatch between EuSi_2_ and Si is large, approximately 12%.

The absence of any intermediate layer at the interface is found in high-resolution (HR) electron microscopy images with different magnification (Fig. 3c,d). This is quite remarkable as an amorphous interlayer is found to occur in most metal/silicon systems^6^. Another observation is the presence of atomic steps on the Si surface with the height varying between *a*_*si*_/2 and 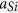, where 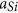 is the Si unit cell constant. Inverse fast Fourier transform images (not presented here) show that the lattice mismatch between EuSi_2_ and Si is released through misfit dislocations. The projection of the Burgers vector on (110)_Si_ is 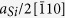. The average distance between dislocations is 20 Å – the value expected from the lattice mismatch between EuSi_2_ and Si. Further inspection of HR images reveals out-of-phase boundaries (OPBs) in the EuSi_2_ film as well as regions of 50–200 Å size with tiny misorientations. The formation of OPBs is associated with the steps at the interface: the shift between the adjacent regions is close to *c*/4. The density of OPBs diminishes from the EuSi_2_/Si interface to the EuSi_2_ surface. The misorientations come from misfit dislocations and related strains. The film homogeneity is established by TEM studies of 8 × 5 μm^2^ specimens taken from different parts of the 25 × 25 mm^2^ EuSi_2_/Si film. A remarkable quality of both the film and the interface manifests marked progress in manufacturing EuSi_2_/Si junction.

**Figure 4:**
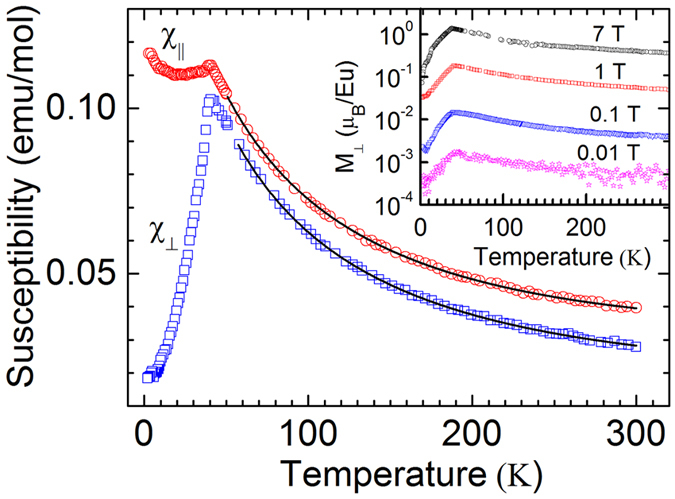
The temperature dependence of the magnetic susceptibility of the EuSi_2_/Si junction measured in magnetic field H = 1 T applied along the surface of the 56 nm film (*χ*_||_, red circles) and normal to the surface of the film (*χ*_⊥_, blue squares). Solid black lines show Curie-Weiss law approximations of *χ*_||_ and *χ*_⊥_ above the Néel temperature. Inset: the temperature dependencies of the magnetization per Eu atom for different magnetic fields normal to the surface of the EuSi_2_ film.

The temperature dependence of magnetic susceptibilities *χ*_*||*_ and *χ*_⊥_ (Fig. 4) demonstrates a typical behavior associated with the AFM transition. The observed magnetic anisotropy is consistent with the AFM magnetic easy axis normal to the film surface. The Néel temperature obtained from the cusp in *χ*(*T*) is 41 ± 2 K. This magnetic ordering temperature is the largest (along with that of GdSi_2_) among RE silicides^46^. To compare with other low-SBH silicides, YbSi_2−x_ does not exhibit any magnetic order down to 1.8 K, while the magnetic ordering temperature of ErSi_2−x_ is 2.8 K^46^. In the paramagnetic region the susceptibility follows the Curie-Weiss law (see Fig. 4). The negative Weiss constant, indicative of the AFM behavior, is −50 ± 10 K. The estimated effective moment per Eu ion is about 9 μ_B_, which is close to the effective moment 7.9 μ_B_ associated with Eu^2+^ ions (spin *S* = 7/2). It corroborates the electron energy loss spectroscopy study which determined Eu in its silicide to be divalent^47^. The magnetic field dependence of the sample magnetization (inset of Fig. 4) is linear for T > 5 K and magnetic field up to 7 T. A robust antiferromagnetism of EuSi_2_ constitutes its additional functional advantage: AFM is accompanied by opening a spin gap which eliminates low-lying spin excitations detrimental to spin coherence. This property may enable efficient spin transport.

**Figure 5:**
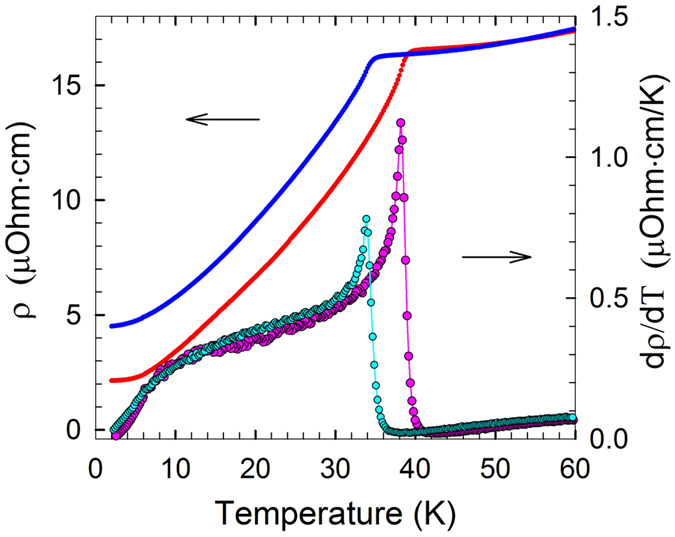
The temperature dependence of the resistivity *ρ*(*T*) and its temperature derivative *dρ*(*T*)/*dT* in zero magnetic field (red and pink curves) and in a magnetic field of 9 T (blue and cyan curves) of the 56 nm EuSi_2_ film.

The transport properties of the films support the results of magnetic measurements. A sharp anomaly associated with the AFM transition is observed in the vicinity of 40 K – the resistivity decreases by an order of magnitude (Fig. 5). The shift of the anomaly in a magnetic field of 9 T is −4.3 K. The resistivity of a magnetic metal is dominated by local spin fluctuations; the temperature dependence of *dρ*(*T*)/dT should follow that of the magnetic specific heat^48^. The form of the *dρ*(*T*)/*dT* curve of the EuSi_2_ films (Fig. 5) does correspond to that for magnetic specific heat in the Ising model for 3D antiferromagnets^49^. Crystalline defects usually suppress the anomaly at the Néel temperature – obviously not in this case. The *dρ*(*T*)/*dT* curve still stays sharp in magnetic field of up to 9 T – it just shifts towards low temperature. Such behavior is a fingerprint of a superb quality of the AFM system.

The resistivity of EuSi_2_ changes from 37 μOhm·cm at room temperature to 2.1 μOhm·cm at 2 K. The residual resistivity is significantly smaller than that observed for ErSi_2_^50^ or ErSi_1.7_^51^ thin films. Hall effect measurements of our films determine the electron concentration about 10^22^ cm^−3^. The Hall mobility increases from 5 cm^2^/(V·s) at room temperature to 500 cm^2^/(V·s) at 1.5 K. These properties make EuSi_2_ a very attractive metallic junction material.

In Si-based nanoelectronics the resistivity of a silicide is coupled with another major characteristic, consumption of Si in its reaction with a metal. Low silicon consumption is a most important technological requirement constraining applications of metal silicides to ultra-shallow junctions and silicon-on-insulator films. Low sheet resistance requires the silicide thickness to be maximized but correspondingly increased Si consumption leads to local junction penetration. It is a major factor that hinders applications of CoSi_2_ and elevates NiSi among other mid-gap silicides. Si consumption is characterized by the ratio of the resulting silicide thickness to the thickness of consumed Si, required to be as large as possible. Typical values are close to 1: 1.10 for TiSi_2_, 0.97 for CoSi_2_, 1.20 for NiSi and 1.27 for stoichiometric ErSi_2_. The same parameter calculated for EuSi_2_ is very large (1.58) making this material highly attractive for nanoscale applications. However, EuSi_2_ is not designed to compete with transition-metal low-resistivity silicides; instead, it is suggested as a prospective material for the SB-MOSFET technology (see below).

**Figure 6:**
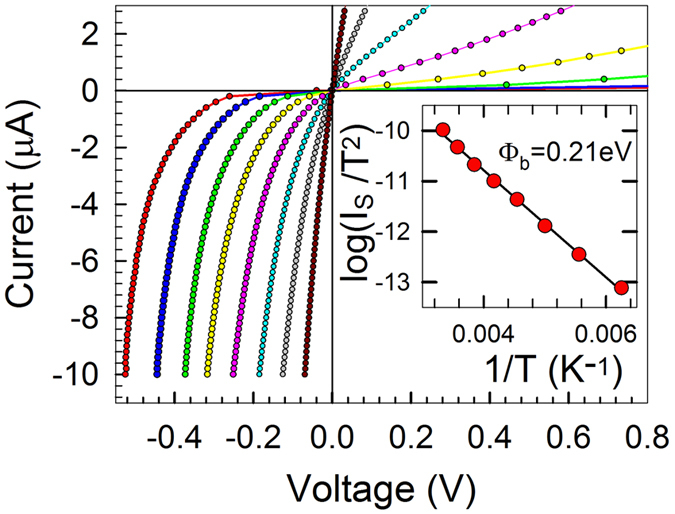
I–V characteristics of the Schottky barrier contact EuSi_2_/*n*-Si measured for a number of temperatures between 160 K (red curve) and 300 K (brown curve) with a step of 20 K. Inset: the linearized temperature dependence of the saturation current 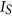 employed to determine the Schottky barrier height.

Injection of carriers into Si from a metal contact is governed by the SBH. The Si substrate in our experiments exhibits resistivity 3.8 kOhm·cm, electron concentration 1.5·10^12^ cm^−3^ and Hall mobility 1100 cm^2^V^−1^s^−1^. The current-voltage characteristic of the SBH for the grown EuSi_2_/*n*-Si contact is distinctly asymmetric in the temperature region between 160 K and 300 K (Fig. 6). With a small forward bias the I–V curve is exponential. Defects at the metal/Si interface effectively increase the SBH and hinder the injection^4^. The I–V curve for the EuSi_2_/*n*-Si contact follows the ideal (not influenced by surface states) classical thermionic theory^52^, yet another indication of the quality of the interface:


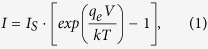



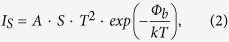


where *I*_*S*_ is the saturation current, *T* – the absolute temperature, *q*_*e*_ – the electron charge, *A* is the Richardson constant, *S* is the contact area and *Φ*_*b*_ is the SBH. According to our data the SBH of the EuSi_2_/*n*-Si junction is 0.21 ± 0.01 eV, significantly smaller than the values known for other RE silicides^16^. The record-low Schottky barrier height constitutes a major advantage of EuSi_2_ over other silicides in competition for employment as contact material in electronics.

In summary, taking into account technological advantages of metal silicides and their full compatibility with Si technology we propose europium disilicide as a prospective junction material. In the course of our work we optimized conditions for manufacturing EuSi_2_/Si contacts. Epitaxial films are grown by reaction of Eu with silicon substrate. The synthesis is robust, easy to implement and what is most important is free from unwanted side products. Moreover, electron microscopy shows that the EuSi_2_/Si interface is atomically abrupt despite a significant lattice mismatch.

Apart from the superb structural quality of the EuSi_2_/Si interface and EuSi_2_ film, europium silicide exhibits a combination of properties which respond to demands of modern electronics: At low temperature EuSi_2_ becomes antiferromagnetic which may assist applications employing spin-related phenomena. Rather low resistivity and very low Si consumption are among other advantages of the material. Most importantly, the EuSi_2_/*n*-Si junction exhibits the lowest among silicides Schottky barrier height. Overall, EuSi_2_ is the most promising material for the SB-MOSFET technology.

## Methods

### Synthesis

The samples are grown in Riber Compact 12 system for molecular beam epitaxy furnished with UHV system comprising Gamma Vacuum Titan Ion Pump, cryopump Cryo-Torr 8 (Brooks CTI Cryogenics), titanium sublimation pump and cryopanels cooled down by liquid nitrogen. The base pressure is less than 10^−10^ Torr. 4N Eu and capping material SiO are supplied from Knudsen cell effusion sources. The temperature of the substrate is controlled with PhotriX ML-AAPX/090 infrared pyrometer (LumaSense Technologies) operating at the 0.9 μm wavelength. Molecular beam intensity is measured with Bayard-Alpert ionization gauge fitted at the substrate site. The substrates are high-ohmic compensated Si (001) wafers with miscut angles not exceeding 0.5°.

### Transmission Electron Microscopy

The cross-sectional samples for analytical TEM/STEM are prepared with 2 different techniques. One is a standard procedure comprising mechanical polishing of cross-sections down to a thickness of 20–25 μm followed by ion milling with Ar^+^ using Gatan 691 PIPS at an accelerating voltage of 3 keV until perforation; the final milling is carried out with 0.1 keV Ar^+^ ions. The other procedure employs Helios (FEI) scanning electron microscope (SEM)/Focus Ion Beam (FIB) dual beam system equipped with gas injectors for C and Pt deposition and a micromanipulator (Omniprobe). A 2 μm Pt layer is deposited on the surface of the sample. FIB milling (30 keV Ga^+^ ions) results in 2 μm thick cross-sections of approximately 8 × 5 μm^2^ area. Electron transparency is achieved by further thinning and final cleaning with 5 keV and 2 keV Ga^+^ ion beams, respectively. The cross-sections are covered by thin C layers to prevent oxidation in the Helios chamber before breaking the vacuum. The specimens are studied with a TEM/STEM Titan 80–300 (FEI) operating at 300 kV. The microscope is equipped with a spherical aberration (C_s_) corrector, a HAADF detector, an atmospheric thin-window energy dispersive X-ray spectrometer (Phoenix System, EDAX) and a post-column Gatan energy filter (GIF). The images are analysed with the Digital Micrograph (Gatan) and Tecnai Imaging and Analysis (FEI) software.

### Characterization

The surface of the films is controlled *in situ* with reflection high-energy electron diffractometer fitted with kSA 400 Analytical RHEED System (k-Space Associates, Inc.). X-ray diffraction data are obtained with Bruker D8 Advance spectrometer (CuK_α_ X-ray source). Magnetization measurements of the films are carried out with MPMS XL-7 SQUID magnetometer (Quantum Design) using reciprocating sample option (RSO). The samples are mounted in plastic straws orienting the surface of the films parallel or perpendicular to the external magnetic field with the accuracy better than 5°. The diamagnetic moment of the Si substrate exceeds the magnetic moment of thin EuSi_2_ films; its subtraction from the signal generates a systematic error of about 10% in the value of magnetization. The demagnetization field is not taken into account. Transport measurements of resistivity and Hall effect in EuSi_2_ and I–V characteristics of the EuSi_2_/Si junction are carried out by the four-terminal sensing method using Lake Shore 9709A Hall effect measurement system.

## Additional Information

**How to cite this article**: Averyanov, D. V. *et al.* Europium Silicide – a Prospective Material for Contacts with Silicon. *Sci. Rep.* **6**, 25980; doi: 10.1038/srep25980 (2016).
